# Highly Expressed Soluble Recombinant Anti-GFP VHHs in *Escherichia coli via* Optimized Signal Peptides, Strains, and Inducers

**DOI:** 10.3389/fmolb.2022.848829

**Published:** 2022-03-10

**Authors:** Shuangying Chao, Yuhang Liu, Ning Ding, Yue Lin, Qian Wang, Junwen Tan, Wei Li, Yang Zheng, Xuejun Hu, Junming Li

**Affiliations:** ^1^ Medical College, Dalian University, Dalian, China; ^2^ Dalian Key Laboratory of Oligosaccharide Recombination and Recombinant Protein Modification, Dalian, China; ^3^ Department of Clinical Laboratory, Yantai Yuhuangding Hospital, Yantai, China

**Keywords:** signal peptide, inducer, *Escherichia coli*, anti-GFP VHHs, inclusion body, soluble protein

## Abstract

Antigen-binding variable domains of the H chain of heavy-chain antibodies (VHHs), also known as nanobodies (Nbs), are of great interest in imaging technique, disease prevention, diagnosis, and therapy. High-level expression of soluble Nbs is very important for its industrial production. In this study, we optimized the expression system of anti-green fluorescent protein (GFP) VHHs with three different signal peptides (SPs), outer-membrane protein A (OmpA), pectate lyase B (PelB), and L-asparaginase II SP (L-AsPsII), in different *Escherichia coli* strains *via* isopropyl β-D-thiogalactoside (IPTG) induction and auto-induction, respectively. The solubility of recombinant anti-GFP VHHs with PelB or OmpA was significantly enhanced to the same extent by IPTG induction and auto-induction in BL21 (DE3) *E. coli* strain and the maximum yield of target protein reached approximately 0.4 mg/l in a shake flask. The binding activity of recombinant anti-GFP VHHs was also confirmed to be retained by native-polyacrylamide gel electrophoresis (PAGE). These results suggest that SPs like OmpA and PelB could efficiently improve the recombinant anti-GFP VHH solubility without changing its bioactivity, providing a novel strategy to optimize the *E. coli* expression system of soluble VHHs, and lay the foundation for the industrial production of soluble recombinant anti-GFP VHHs and the research of other VHHs in the future.

## Introduction

Recombinant protein production *via* prokaryotic system has brought hundreds of therapeutic proteins into clinical applications or clinical trials over the past few decades (Dimitrov, 2012; Sánchez-Trasviña et al., 2021). Recombinant proteins are frequently soluble. In some cases, when high-level expression of recombinant proteins in *Escherichia coli* occurs, insoluble aggregates accumulate as inclusion bodies. Numerous strategies were attempted to overcome this problem, such as the optimization of bacterial strains, promoters, inducers, as well as the use of N-terminal signal peptides (SPs), which could help recombinant proteins be secreted into the culture medium (Tegel et al., 2011; Chen, 2012; Freudl, 2018; Owji et al., 2018; Liu et al., 2019). Compared to intracellular expression, extracellular production of proteins possesses several advantages. First, extracellular expression could prevent aggregations of proteins and accumulation of inclusion bodies. Second, protein secretion from cytoplasm to periplasm provides an oxidative environment, which facilitates correct protein folding and disulfide bond formation (Ruiz et al., 2006; Mergulhão and Monteiro, 2007; Silhavy et al., 2010; Rosano and Ceccarelli, 2014; Kleiner-Grote et al., 2018; Gonzalez-Perez et al., 2021), which is essential for their biological activities. Moreover, secretion of proteins into growth medium requires no cell disruption as well as downstream purification steps, reducing the complexity of bioprocess and significantly improving product purity since there is no intracellular protein contamination (Kleiner-Grote et al., 2018; Green et al., 2019; Yao et al., 2019; Gonzalez-Perez et al., 2021). Therefore, a variety of strategies have been taken to accomplish extracellular expression (Ni and Chen, 2009).

Proteins are generally exported via the general secretory (Sec) pathway (Costa et al., 2015; Burdette et al., 2018), which commonly exists in prokaryotes and eukaryotes (Cao and Saier, 2003). Hundreds of Sec-type SPs were screened in bacteria (Brockmeier et al., 2006; Fu et al., 2018), and several best-performing SPs were selected, such as outer-membrane protein A (OmpA) (Jo et al., 2014; Pechsrichuang et al., 2016), pectate lyase B (PelB) (Wang et al., 2016; Deb et al., 2017; Kulmala et al., 2019), as well as L-asparaginase II SP (L-AsPsII) (Tan et al., 2002; Ismail et al., 2011). However, a SP efficient for one target protein may not be suitable for another (Zamani et al., 2016). Therefore, the optimization of SPs for each target protein is still needed.

Antigen-binding variable domains of the H chain of heavy-chain antibodies (VHHs), also known as nanobodies (Nbs), are functionally equivalent to the Fab fragment of traditional antibodies and possess high affinities to their antigens (Muyldermans, 2013). The Nbs against the green fluorescent protein (GFP), anti-GFP VHHs, have been an extraordinary useful tool for imaging technique, such as single-molecule super-resolution microscopy (Ries et al., 2012; Szymborska et al., 2013; Platonova et al., 2015). In order to efficiently express the soluble anti-GFP VHHs, it is necessary to determine the best induction condition of anti-GFP VHHs.

In this study, we investigated the expression levels of both soluble and insoluble anti-GFP VHHs with three SPs mentioned above (OmpA, PelB, and L-AsPsII) in four *E. coli* strains, BL21 (DE3), Origami2 (DE3), HMS174 (DE3), and ArcticExpress (DE3), *via* isopropyl β-D-thiogalactoside (IPTG) induction and auto-induction, respectively. The better-performing condition of expression was explored, suggesting these approaches could produce highly soluble recombinant anti-GFP VHHs.

## Materials and Methods

### Bacterial Strains, Plasmids, and Growth Conditions

The *E. coli* strain BL21 (DE3) used in this study was purchased from Sangon Biotech (Shanghai); Origami 2 (DE3) and ArcticExpress (DE3) were purchased from Beijing Zoman Biotechnology (Beijing); and HMS174 (DE3) was purchased from Beyotime Biotechnology (Shanghai). The anti-GFP VHHs named A12 was used as the model protein in our research (Fang et al., 2020). The expressing plasmids named pCL were synthesized based on the sequence of pIG6 (Zhu et al., 2020); the Kan resistance gene (Ruan et al., 2021) was taken to replace the Amp resistance gene in this new vector; and the expression cassette was constructed in the form T7 promoter-leader peptide-anti-GFP gene and T7 terminator. The anti-GFP gene carried OmpA, PelB, and L-AspsII signal peptides, respectively. Strains were grown on LB agar plates and in LB broth (10 g tryptone, 5 g yeast extract, and 10 g NaCl in 400 ml water) supplemented with kanamycin (50 μg/ml), which was used for IPTG induction. ZY broth [1 M MgSO_4_, 50 × 5,052, 20× NPS, and ZY (10 g tryptone and 5 g yeast extract per liter)] (William Studier, 2005) supplemented with kanamycin (50 μg/ml) was used for auto-induction.

### Expression of Recombinant Anti-GFP VHHs

The expression of recombinant anti-GFP VHHs with different signal peptide was driven by a T7 promoter. *E. coli* BL21 (DE3), Origami2 (DE3), ArcticExpress (DE3), and HMS174 (DE3) cells were transformed with the different expression vectors. For protein expression, a single colony was selected and cultured in 3-ml LB media supplemented with 50 μg/ml of kanamycin. The overnight culture of BL21 (DE3) cells containing the expression vector was used to inoculate fresh LB media with 50 μg/ml of kanamycin in a 1:1,000 dilution; then, the cultures were shaken at 220 rpm at 37°C until OD_600_ reached 0.6–0.8. Protein expression was induced by the addition of 0.2 mM of IPTG for IPTG induction or by inoculation of a fresh ZY medium with 50 μg/ml of kanamycin in a 1:1,000 dilution for auto-induction at 25°C with 220 rpm shaking. To determine the best post-induction time, two OD_600_ cultures were collected at various time points after induction and pelleted by centrifugation at 12,000 rpm, at 4°C for 10 min. For purification, cells were harvested from 500 ml of ZY medium after 24 h of auto-induction by centrifugation at 11,000 rpm, at 4°C for 10 min. Cell pellets were stored at −80°C until further use.

### SDS-PAGE Detection and Quantification of Soluble/Insoluble Fractionation of Anti-GFP VHHs

Cell pellets from per OD_600_ were re-suspended in 80 μl of ice-cold phosphate buffer (PB). Then, the cells were sonicated on ice for 10 × 5-s bursts, with 5-s cooling in between, at an amplitude of 30 microns. The lysate was collected and centrifuged at 12,000 rpm, at 4°C for 20 min. The supernatant was recovered as the “soluble” fraction. After washing once by ice-cold PB, the pellets were re-suspended in 80 μl of ice-cold PB as “insoluble” fraction. All samples were mixed with 20 μl of 5× loading buffer separately and boiled for 10 min. After cooling to room temperature, 6-μl samples were separated by 15% polyacrylamide gel at 120 V for 1 h, 20 min. The gels were stained with Coomassie brilliant blue R-250 to detect protein and analyzed by using a gel imaging analysis system (Tanon 2500R). The protein lane corresponding to the soluble and insoluble protein at the last time point was quantified by bicinchoninic acid (BCA) assay with bovine serum albumin (BSA) as standard, and the target protein bands were relatively quantified by grayscale scanning with ImageJ.

### Purification of Recombinant Anti-GFP VHHs

The yield of recombinant anti-GFP VHHs with PelB expressed in BL21 (DE3) *via* auto-induction was higher than others, so we chose PelB and BL21 (DE3) as the best signal peptide and expressing strain, respectively. The cell pellet from 400-ml expression cultures was re-suspended in 20-ml ice-cold PB ( PB, 0.02 M KH_2_PO_4_, and 0.02 M Na_2_HPO_4_, pH7.5). After repeated freezing and thawing twice, lysozyme was added to the bacterial suspension to achieve a final concentration of 50 μg/ml, at 37°C water bath for 30 min. Cells were lysed by sonication on ice for 20 min of 3-s pulse with 2-s intervals at 200 W. The supernatant was collected by centrifugation at 12,000 rpm, at 4°C for 10 min. Then, 5 mg/ml DNase I was added into the supernatant to achieve a final concentration of 50 μg/ml, which was then suspended in an ice bath for 20 min. Samples were cleared by centrifugation at 12,000 rpm, at 4°C for 10 min. An equal volume of 40-mM imidazole was added to the supernatant to achieve a final concentration of 20 mM before filter sterilization. Tween-20 was added into the supernatant with a final concentration of 2% prior to affinity purification. The anti-eGFP VHH protein was purified by a HisTrap Chelating HP column (GE Healthcare) charged with Ni^2+^ ions. The column was pre-equilibrated with binding buffer (0.02 M PB and 0.5 M NaCl, pH7.5). Following loading of the sample and washing the column with washing buffer (0.02 M PB, 0.5 M NaCl, and 20 mM imidazole, pH7.5), elution buffer with different concentrations of imidazole (0.02 M PB; 0.5 M NaCl; and 40, 80, 120, 240, or 500 mM imidazole, pH7.5) was added to the column in order to elute the target protein. All samples were analyzed using SDS-PAGE (20 μl/sample).

Protein concentrations were measured using the BCA assay with BSA as standard.

### Western Blot Detection of Anti-GFP VHHs

The samples corresponding to the highest expression level of each signal peptide in BL21 (DE3) were mixed with 20 μl 5× loading buffer separately and boiled for 10 min. After cooling to room temperature, 3-μl samples were separated by 15% polyacrylamide gel and transferred onto a polyvinylidene fluoride (PVDF) membrane. Following blocking of non-specific binding sites with 3% skim milk in TBST buffer (20 mM Tris-HCl pH 8.0, 150 mM NaCl, 0.05% Tween-20) overnight at 4°C, the PVDF membrane was further incubated with mouse anti-His antibody (Invitrogen, United States) diluted 1:4,000 in TBST buffer for 1 h at room temperature. The membrane was then washed 8 × 5 min in TBST buffer prior to 30-min incubation with the secondary antibody conjugated to horseradish peroxidase (Thermo Fisher Scientific, United States) diluted 1:4,000 in TBST buffer. An enhanced chemiluminescence kit (Shanghai Epizyme Biomedical Technology Co., Ltd., China) and the system with Image Lab^TM^ image capture software (Bio-Rad, United States) were used for signal detection.

In order to quantitate the percentage of soluble and insoluble protein, we added a known amount of VHH quantified by BCA as a reference. ImageJ was used for the intensity analysis. The soluble and insoluble proteins were quantified according to the VHHs that have been quantified. The sum of soluble and insoluble proteins was reported as the total protein.

### Native-PAGE

The purified anti-GFP VHHs were incubated with ELP30-eGFP protein at 37°C for 40 min at equal moles, supplemented with the equal volume of sample buffer (100 mM Tris-HCl, pH 6.8, 20% (*v*/*v*) glycerol, and 0.2% (*m*/*v*) bromophenol blue), and then separated in a 12% polyacrylamide gel prepared in electrophoresis buffer (25 mM Tris and 0.2 M glycine). Native-PAGE was performed for 8 h on ice (100 V), following which the gel was stained with Coomassie brilliant blue (CBB).

## Results

### The Expression of Anti-GFP VHHs With Different Signal Peptides *via* IPTG Induction

Since it was reported that several anti-GFP VHH mutants were found to possess enhanced binding activities by changing their amino acid sequences of the complementarity determining region 3 (CDR3) *via* phage display technique (Fang et al., 2020), we used the best-performing mutants as the model recombinant VHHs in this work. Plasmids containing the sequences of VHHs each with SP or without SPs were constructed, and the amino acid sequences of each SP are shown in Table 1.

**TABLE 1:** Amino acid sequence of signal peptides used in this study.

Signal peptides (SPs)			Plasmids	VHH name
No.	Name	Amino acid sequence		
1	OmpA	MKKTAIAIAVALAGFATVAQA	pCL-OmpA-anti-GFP VHHs	OmpA-VHHs (17.8 kD)
2	PelB	MKYLLPTAAAGLLLLAAQPAMA	pCL-PelB-anti-GFP VHHs	PelB-VHHs (18.0 kD)
3	L-AsPsII	MEFFKKTALAALVMGFSGAALA	pCL-L-AsPsII-anti-GFP VHHs	L-AsPsII-VHHs (18.1 kD)
4	N/A	N/A	pCL-anti-GFP VHHs	VHHs (15.8 kD)

**FIGURE 1:**
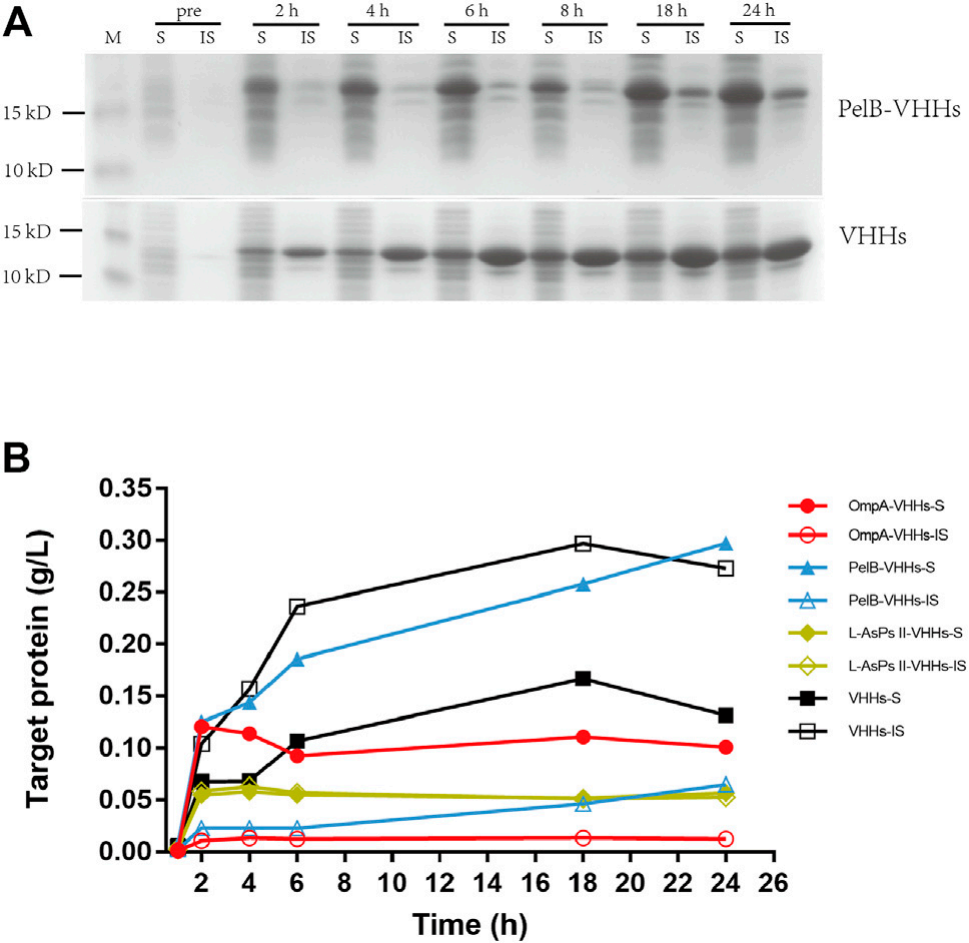
The expression of anti-green fluorescent protein (GFP) variable domains of the H chain of heavy-chain antibodies (VHHs) with different signal peptides (SPs) in isopropyl β-D-thiogalactoside (IPTG)-induced *E. coli* BL21 (DE3). **(A)** sodium dodecyl sulfate–polyacrylamide gel electrophoresis (SDS-PAGE) of VHHs with PelB (upper panel) and VHHs alone (lower panel). **(B)** The time-dependent expression level of VHHs alone (black) and VHHs with OmpA (red), PelB (blue), and L-AsPs II-SP (yellow). S, soluble fraction; IS, insoluble fraction. Concentration of IPTG: 0.02 mM. Induction temperature: 25°C. M: Enhanced 3-color Regular Range SMOBIO Protein Marker (PA2511) (10.0–180 kDa) purchased from SMOBIO.

**FIGURE 2:**
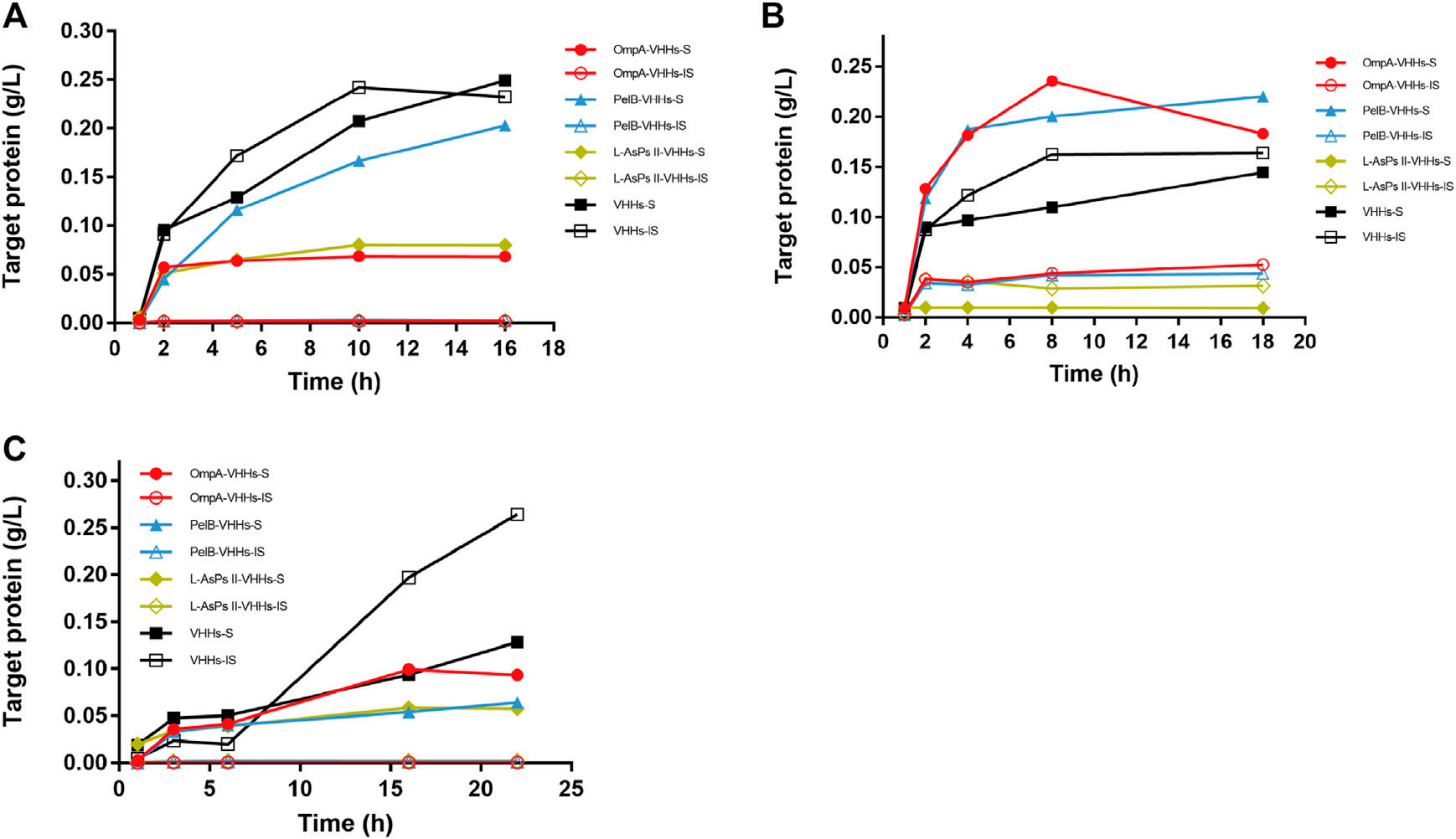
The expression of anti-GFP VHHs with different SPs in IPTG-induced *E. coli* Origami2 (DE3) **(A)**, ArcticExpress (DE3) **(B)**, and HMS174 (DE3) **(C)**. The time-dependent expression level of VHHs alone (black) and VHHs with OmpA (red), PelB (blue), and L-AsPs II-SP (yellow). S, soluble fraction; IS, insoluble fraction. Concentration of IPTG: 0.02 mM. Induction temperature: 25°C. M: Enhanced 3-color Regular Range SMOBIO Protein Marker (PA2511) (10.0–180 kDa) purchased from SMOBIO.

Each plasmid was transformed into BL21 (DE3), and the protein expression was induced by IPTG. The soluble and insoluble portions of total protein at different induction time points were checked by sodium dodecyl sulfate (SDS)-PAGE analysis. The result showed that PelB significantly increased the soluble form of VHHs in a time-dependent manner, and the soluble PelB-VHH expression was approximately two-fold higher than soluble VHHs without SPs (Figure 1), while OmpA and L-AsPsII did not improve the solubility of VHHs (Figure 1B and Supplementary Figure S1). We then transformed these plasmids into three other *E. coli* strains and performed the same procedures. Intriguingly, all three SPs including PelB did not increase the amount of soluble VHHs but clearly reduced that of insoluble VHHs in Origami2 (DE3) and HMS174 (DE3) (Figure 2A, C and Supplementary Figures S2, S4). The soluble PelB-VHHs showed a slight increase in ArcticExpress (DE3) (Figure 2B and Supplementary Figure S3B), which was similar to the result of BL21 (DE3). Additionally, OmpA also slightly facilitated the solubility of VHHs in ArcticExpress (DE3) (Figure 2B and Supplementary Figure S3A).

### The Expression of Anti-GFP VHHs With Different Signal Peptides *via* Auto-Induction

**FIGURE 3:**
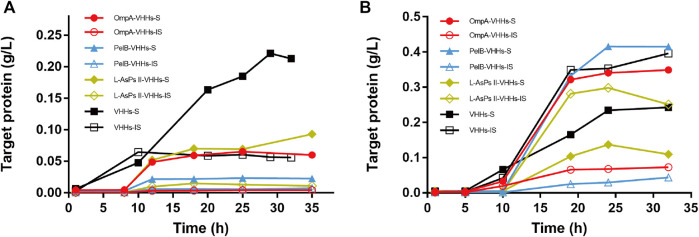
The expression of anti-GFP VHHs with different SPs *via* auto-induction in *E. coli* Origami2 (DE3) **(A)** and BL21 (DE3) **(B)**. The time-dependent expression level of VHHs alone (black) and VHHs with OmpA (red), PelB (blue), and L-AsPs II-SP (yellow). S, soluble fraction; IS, insoluble fraction. Concentration of IPTG: 0.02 mM. Induction temperature: 25°C. M: Enhanced 3-color Regular Range SMOBIO Protein Marker (PA2511) (10.0–180 kDa) purchased from SMOBIO.

The expression of total protein *via* auto-induction was also checked by SDS-PAGE analysis. However, almost no recombinant VHH was expressed in HMS174 (DE3) and ArcticExpress (DE3) (data not shown). In Origami2 (DE3), all three SPs could not improve the solubility of VHHs (Figure 3A and Supplementary Figure S6). However, PelB slightly increased the solubility of VHHs in BL21 (DE3), and the maximum yield of target protein reached 400 mg/l (Figure 3B and Supplementary Figure S5B). Intriguingly, OmpA could increase the solubility of VHHs *via* auto-induction in BL21 (DE3), which was not observed in IPTG-induced BL21 (DE3) (Figure 3B and Supplementary Figure S5A).

### Western Blot Analysis of Soluble and Insoluble Anti-GFP VHHs With Different Signal Peptide *via* IPTG Induction and Auto-Induction

**FIGURE 4:**
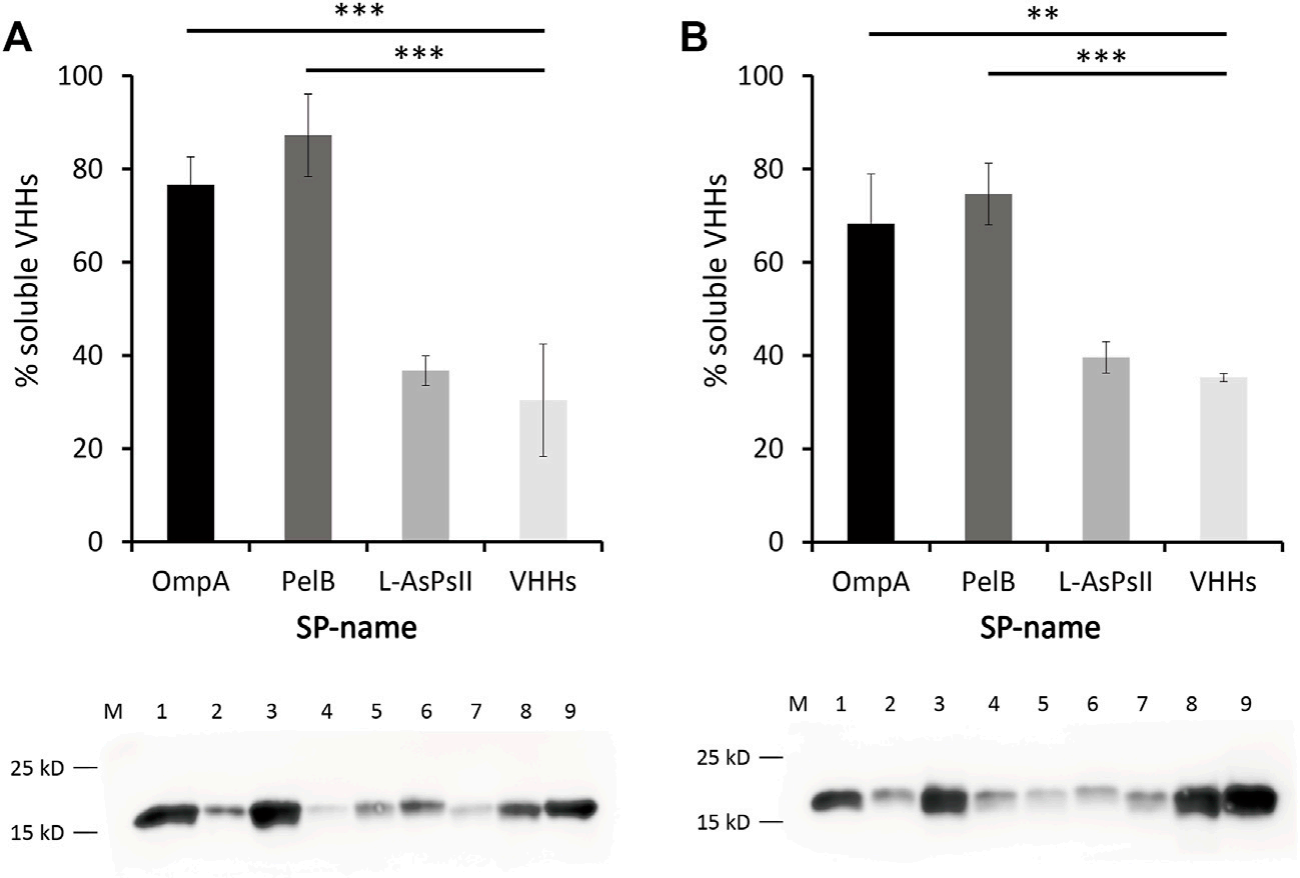
Western blot analysis of anti-GFP VHHs with different SPs in auto-induced **(A)** and IPTG-induced *E. coli* BL21 (DE3) **(B)**. Percentage of soluble VHHs relative to the total VHHs is shown (upper panel). Proteins were quantified by a pre-determined purified VHHs. ImageJ was used for the intensity analysis. The soluble and insoluble proteins were quantified according to the VHHs that have been quantified *via* bicinchoninic acid (BCA) assay. The sum of soluble and insoluble protein was reported as the total protein. Western blot of VHHs alone and VHHs with different SPs is shown (lower panel). M: maker, lane 1: OmpA-VHHs-S, lane 2: OmpA-VHHs-IS, lane 3: PelB-VHHs-S, lane 4: PelB-VHHs-IS, lane 5: L-AsPs II-VHHs-S, lane 6: L-AsPs II-VHHs-IS, lane 7: VHHs-S, lane 8: VHHs-IS, lane 9: purified VHHs (1.105 μg/μl, positive control). S, soluble fraction; IS, insoluble fraction. Values are means ± S.D. *n* = 3. The significance of the differences was determined by Tukey’s multiple comparisons test (**p < 0.01 and ***p < 0.001).

Due to the capability of BL21 (DE3) for soluble VHH production by IPTG and auto-induction, western blot assay was further carried out to determine the expression level of soluble and insoluble VHHs in BL21 (DE3). The samples for each SP were collected at the induction time point when the maximum VHH expression was reached. The percentages of soluble VHHs relative to total VHH expression revealed that PelB significantly improved the solubility of VHHs in both IPTG-induced and auto-induced BL21 (DE3), which was consistent with the results of SDS-PAGE (Figure 4). Although OmpA increased the solubility of VHHs only in auto-induced BL21 (DE3) as seen in SDS-PAGE, western blotting data exhibited that it could also facilitate the solubility of VHHs in IPTG-induced BL21 (DE3) (Figure 4). Additionally, no significant difference between IPTG induction and auto-induction was observed for the solubility improved by PelB or OmpA.

### Binding Activity Assay for Anti-GFP VHHs

**FIGURE 5:**
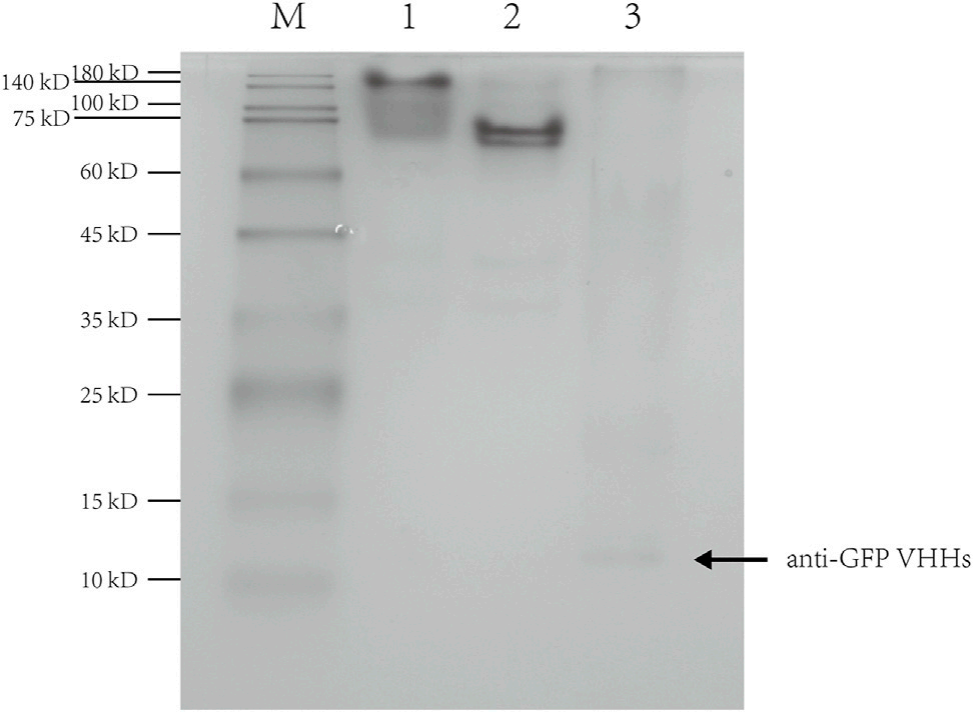
Native-PAGE of the interaction between anti-GFP VHHs and ELP30-GFP. Lane 1: the mixture of anti-GFP VHHs and ELP30-GFP; lane 2: ELP30-GFP alone; lane 3: anti-GFP VHHs alone. M: Enhanced 3-color Regular Range SMOBIO Protein Marker (PA2511) (10.0–180 kDa) purchased from SMOBIO.

To confirm that the anti-GFP VHHs used in this study retained the binding activity to GFP, purified anti-GFP VHHs and GFP with an elastin-like polypeptide tag (ELP30-GFP) were premixed and incubated in 1:1 M ratio, followed by loading into a native gel. The mixture of anti-GFP VHHs and ELP30-GFP showed a significant shift in the banding pattern with a slight streaking (Figure 5). This result suggested a strong interaction between anti-GFP VHHs and ELP30-GFP.

## Discussion

In this study, we constructed four expression plasmids based on pIG6, which were designed to express anti-GFP VHHs alone or with three different SPs including OmpA, PelB, and L-AsPsII. The plasmids were transformed into *E. coli* strain BL21 (DE3), Origami2 (DE3), HMS174 (DE3), as well as ArcticExpress (DE3) (Table 2), followed by IPTG and auto-induction, respectively. However, we observed a low target protein expression in HMS174 (DE3) and ArcticExpress (DE3) *via* auto-induction (data not shown). Origami2 (DE3) was engineered to efficiently form disulfide bonds in the cytoplasm. Anti-GFP VHHs used in this work retained the feature that could form an intermolecular disulfide bridge between CDR1 and CDR3 like other VHHs (Muyldermans, 2001; Fang et al., 2020). Hence, we observed that VHHs produced in auto-induced Origami2 (DE3) almost entirely existed in soluble form even without SPs (Supplementary Figure S6D). In contrast, IPTG-induced Origami2 (DE3) showed a reduced solubility of its counterpart (Supplementary Figure S2D). Since it takes a certain period of time to form disulfide bond, the rapid expression by IPTG induction might lead to an insufficient formation of disulfide bridges, and similar problems were also observed in other post-translational modifications of recombinant proteins such as glycosylation (Zhu et al., 2020). Another advantage of auto-induction is that it usually has much higher production of recombinant proteins compared to IPTG induction due to its slow and long-lasting induction. Since the main purpose of this work is to increase the proportion of soluble VHHs but not the absolute yield of proteins, we did not further prolong the induction time for auto-induction.

**TABLE 2:** The genotypes of the four strains used in this study.

Name	Genotype
BL21 (DE3)	F-ompT hsdSB(rB- mB-)gal dcm (DE3)
Origami2 (DE3)	△(ara-leu)7697 △lacX74△phoA PvuII phoR araD139 ahpC galE galK rpsL F' [lac + lacIqpro](DE3)gor522::Tn10trxB (StrR, Tet^R^)
ArcticExpress (DE3)	*E.coli*, B F-ompT hsds (rB- mB-) dcm^+^ Tet^R^ gal λ(DE3) endA Hte [cpn10cpn60 Gent^R^]
HMS174 (DE3)	K-12, F-,λDE3 [*lacI*,*lac*UV5-T7*gene*1,*ind*1,*sam*7,*nin*5],*rec*A1,*hsd*R19,IN(*rrn*D-*rrn*E)1,*rph-*1,*rpo*B331 (rifR)

We also observed that OmpA and PelB could significantly increase the solubility of VHHs *via* either IPTG induction or auto-induction, and BL21 (DE3) seemed to be a preferred option for production of soluble VHHs rather than Origami2 (DE3). These results were consistent with the previous reports that OmpA and PelB are conventional favored SPs for improving solubility of numerous recombinant proteins (Choi and Lee, 2004). However, the alignments showed that OmpA shares low DNA and amino acid sequence with PelB, suggesting that enhancing solubility of target protein could not be promised by a certain amino acid sequence of SPs (Sletta et al., 2007). Although OmpA and PelB were found to significantly improve the expression of soluble anti-GFP VHHs, there are still numerous SPs that were not investigated in this study. Therefore, it is possible that the production of soluble anti-GFP VHHs could be further improved by finding better-performing SPs than OmpA and PelB. It is well known that Sec-type SPs generally consist of three specific domains: a positively charged N-region, a hydrophobic H-region, and a C-region that contains the cleavage site of signal peptidase (Owji et al., 2018). Recently, several reports found that site-directed mutagenesis of L-AsPsII SP in its N-region and H-region increased the soluble target recombinant protein expression (Ismail et al., 2011; Ismail and Illias, 2017). These results may explain the unsatisfactory performance of native L-AsPsII used in this work and revealed a possibility that mutagenesis of amino acid sequence could also improve other SPs’ performance. Fusing recombinant proteins to the C-terminus of secretory protein such as YebF and MBP was also attempted to improve solubility of target proteins (Zhang et al., 2006; Chen et al., 2012). However, compared to SPs, these secretory proteins may have an inherent disadvantage, which is their steric hindrance. Secretory proteins possess larger molecular mass and cannot be cleaved like SPs, which are generally processed by signal peptidase. Thus, the steric hindrance induced by secretory proteins could significantly inhibit the activities and functions of target recombinant proteins.

## Conclusion

This work presents an attempt to optimize the expression system of anti-GFP VHHs by evaluating different SPs, *E. coli* strains, and other induction conditions. We highly expressed soluble recombinant anti-GFP VHHs in *E. coli*, and the maximum yield of target protein reached 400 mg/l in shake-flask culture, suggesting much higher yield can be promised by the industrial fed-batch fermentation. While many better-performing SPs remain to be explored, our work provides a novel insight on how to efficiently produce the recombinant VHHs with high solubility.

## Data Availability

The original contributions presented in the study are included in the article/Supplementary Material; further inquiries can be directed to the corresponding authors.
