# Supplementary material for: Highly Expressed Soluble Recombinant Anti-GFP VHHs in Escherichia coli via Optimized Signal Peptides, Strains, and Inducers
Source: Front Mol Biosci. 2022 Mar 10;9:848829. doi: 10.3389/fmolb.2022.848829 (PMC8960375; doi:10.3389/fmolb.2022.848829)
Supplement: Supplementary file 1 [file DataSheet1.docx]

Supplementary Material

# Amino acid sequence of anti-GFP named A12.

# MADVQLQESGGGLVQAGGSLRLSCAASGGTFSILSLGWFRQAPGKEREFVAAISRSEGSTDYADFVKGRFMISRENAKNTAYLQMNSLKPEDTAVYFCAASYARRLSTTASRVLYWGQGTQVTVSSQGGGGSKDQNATKSHHHHHH*

1. **Expressing plasmid used in this research.**


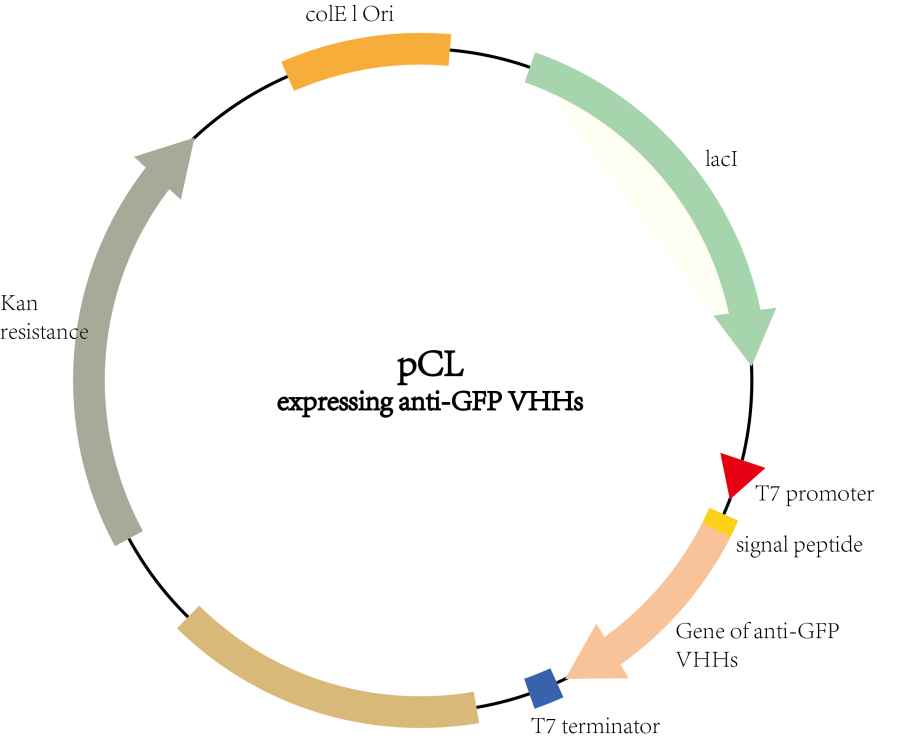


# Supplementary figures


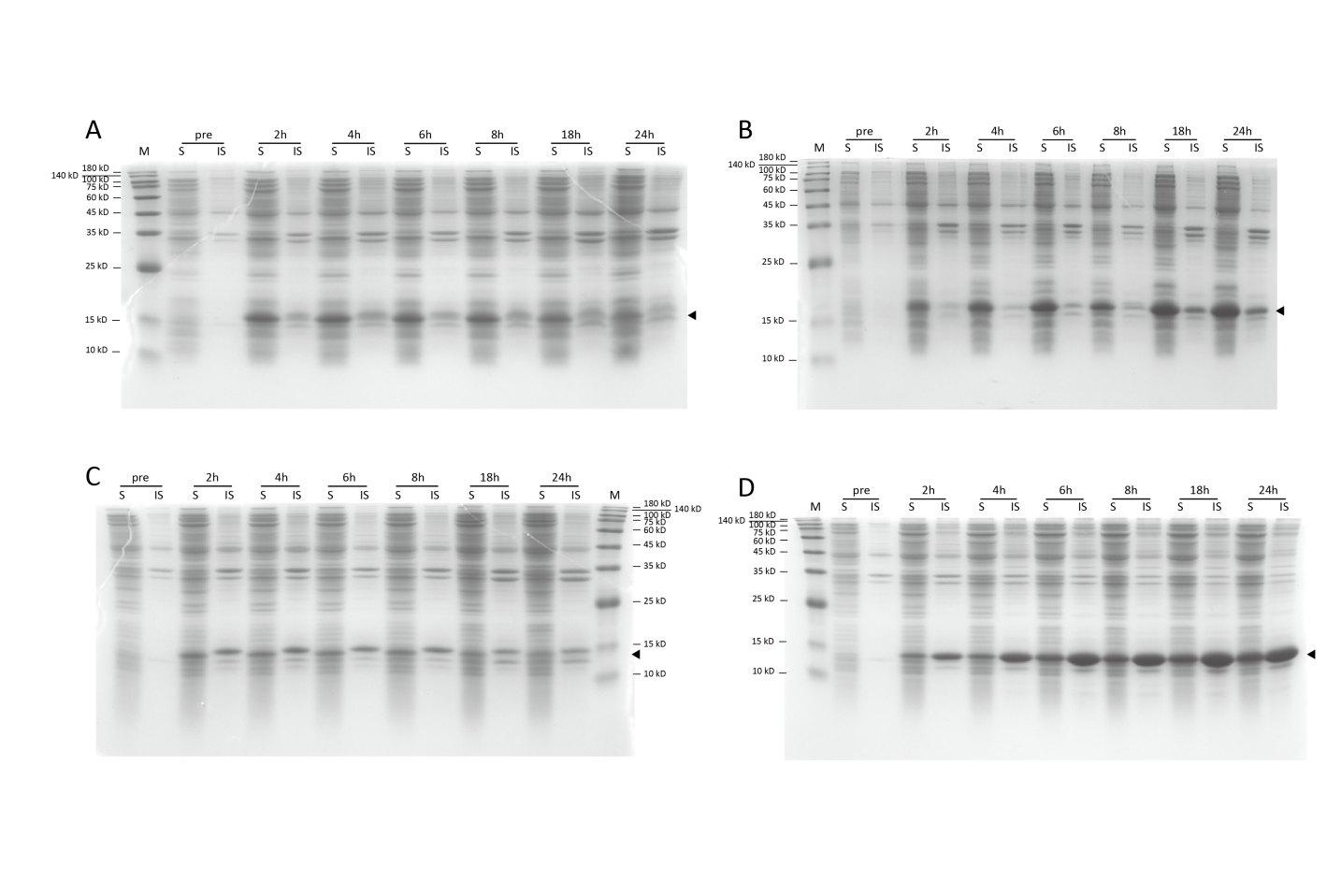


**Supplementary Figure 1.** The expression of anti-GFP VHHs with different SPs in *E.coli* BL21(DE3) via IPTG induction. (A) OmpA, (B) PelB, (C) L-AsPs II, (D) VHHs alone. S: soluble fraction; IS: insoluble fraction. The arrow heads pointed the position of anti-GFP VHHs.


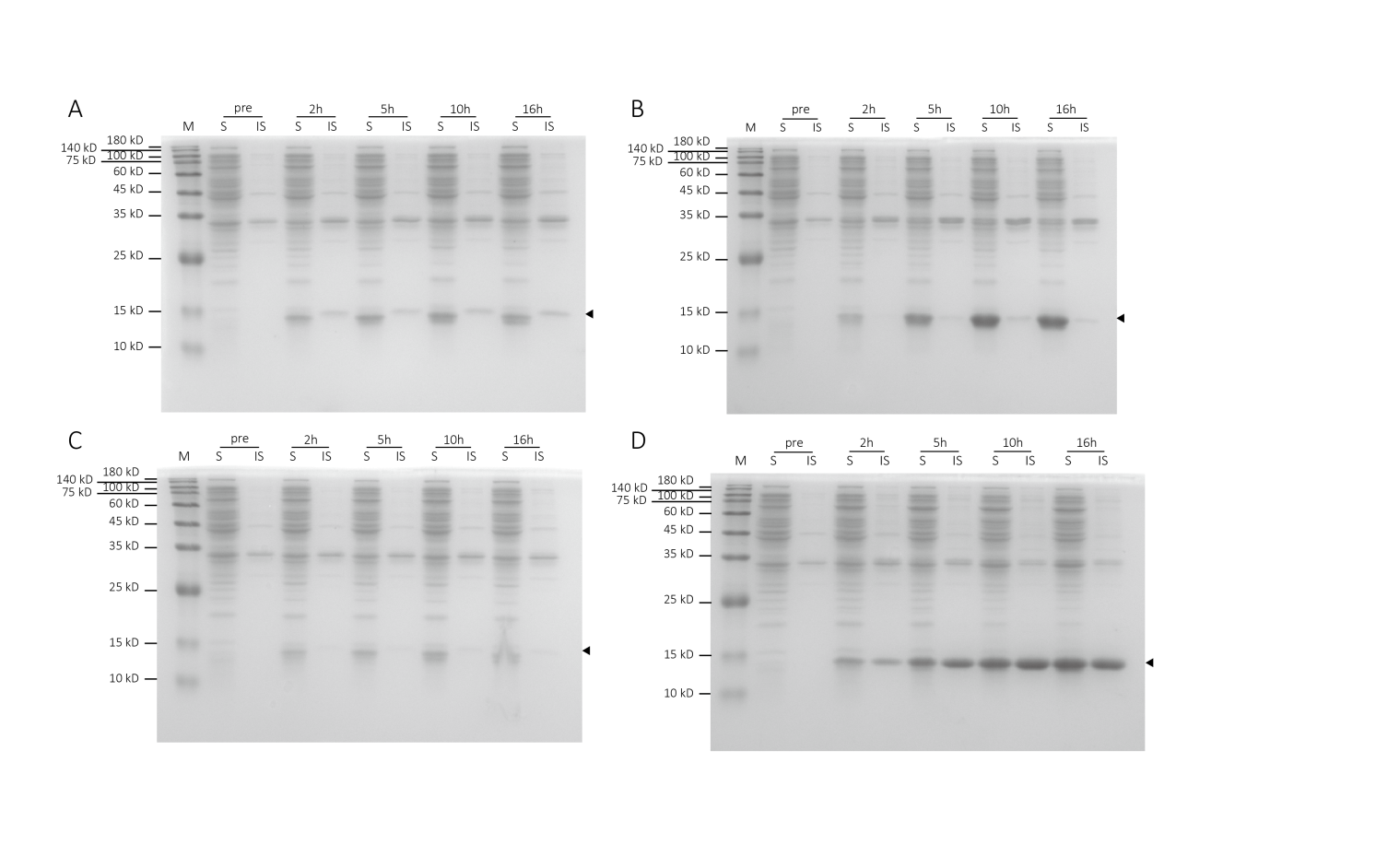


**Supplementary Figure 2.** The expression of anti-GFP VHHs with different SPs in *E.coli* Origami2 (DE3) via IPTG-induction. (A) OmpA, (B) PelB, (C) L-AsPs II, (D) VHHs alone. S: soluble fraction; IS: insoluble fraction. The arrow heads pointed the position of anti-GFP VHHs.


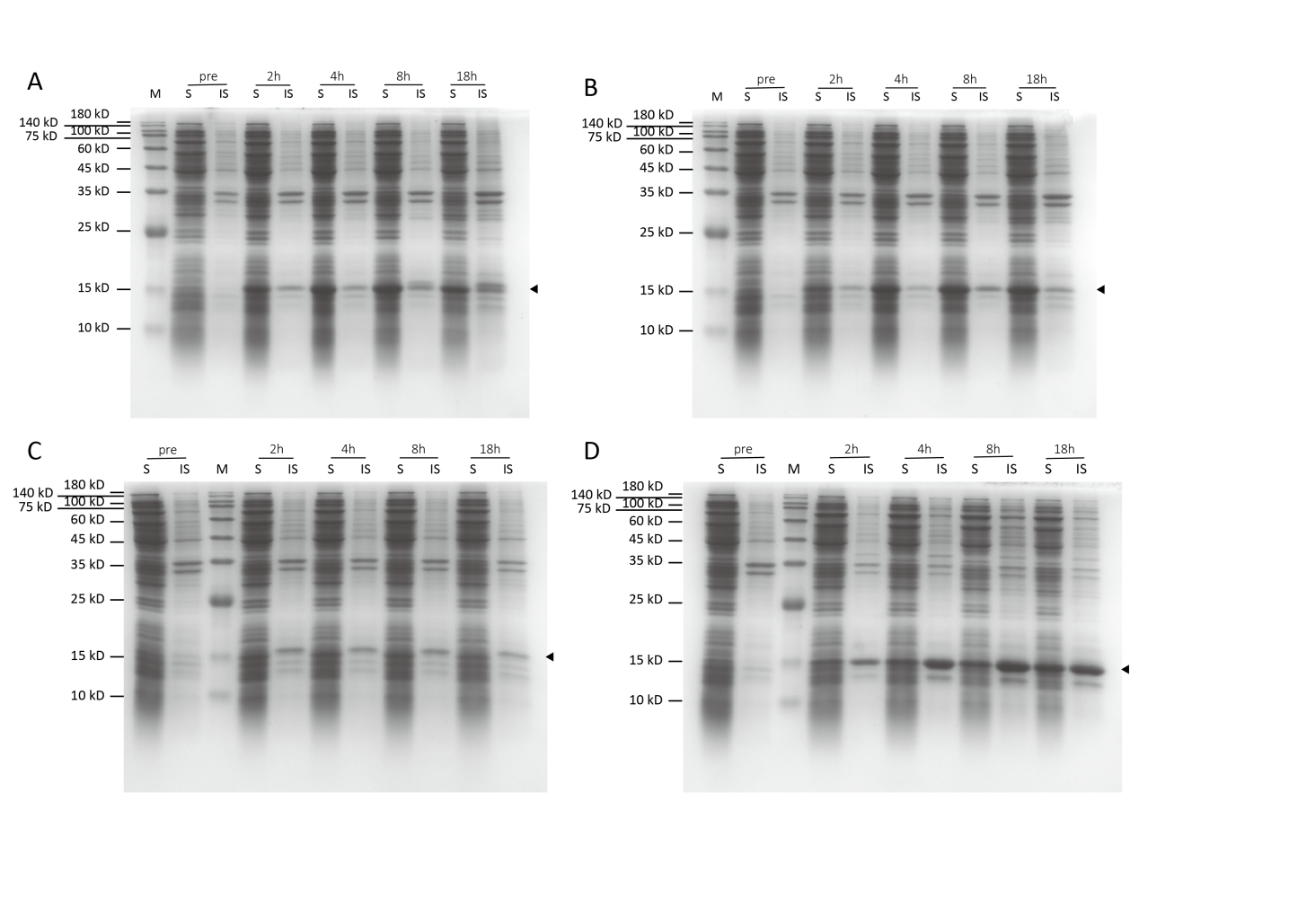


**Supplementary Figure 3.** The expression of anti-GFP VHHs with different SPs in *E.coli* ArcticExpress (DE3) via IPTG-induction. (A) OmpA, (B) PelB, (C) L-AsPs II, (D) VHHs alone. S: soluble fraction; IS: insoluble fraction. The arrow heads pointed the position of anti-GFP VHHs.


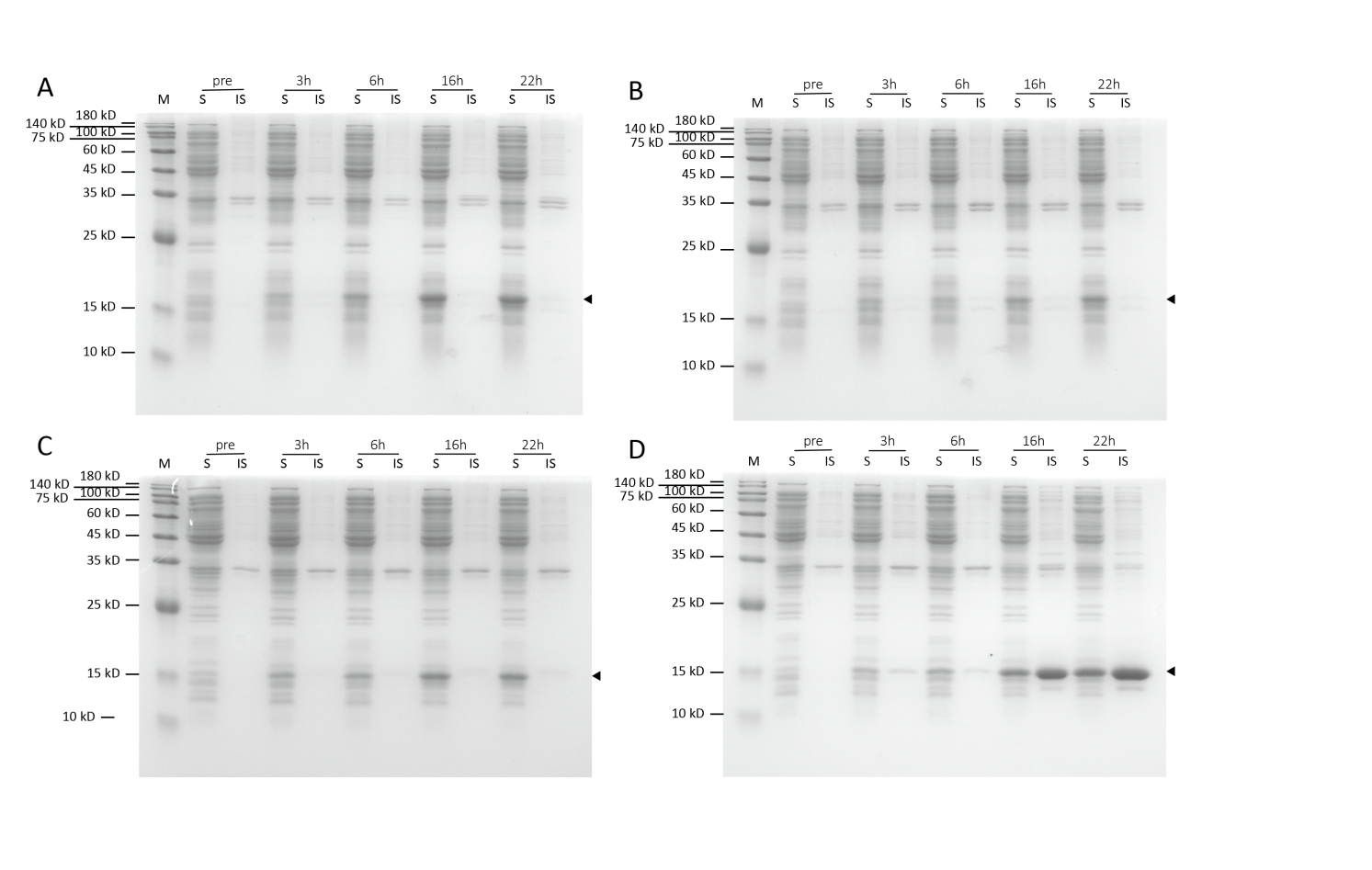


**Supplementary Figure 4.** The expression of anti-GFP VHHs with different SPs in *E.coli* HMS174 (DE3) via IPTG-induction. (A) OmpA, (B) PelB, (C) L-AsPs II, (D) VHHs alone. S: soluble fraction; IS: insoluble fraction. The arrow heads pointed the position of anti-GFP VHHs.


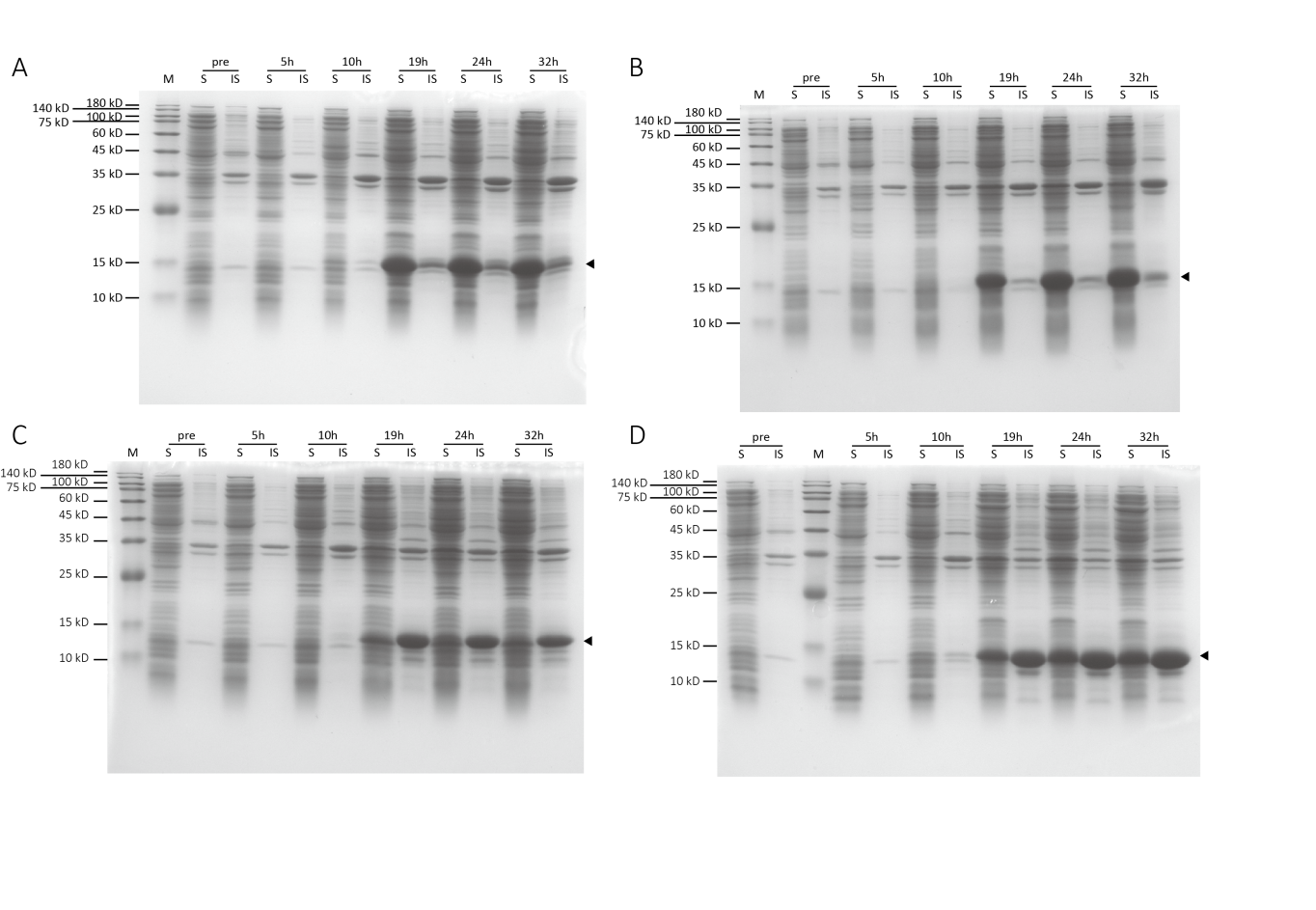


**Supplementary Figure 5.** The expression of anti-GFP VHHs with different SPs in *E.coli* BL21(DE3) via auto-induction. (A) OmpA, (B) PelB, (C) L-AsPs II, (D) VHHs alone. S: soluble fraction; IS: insoluble fraction. The arrow heads pointed the position of anti-GFP VHHs.


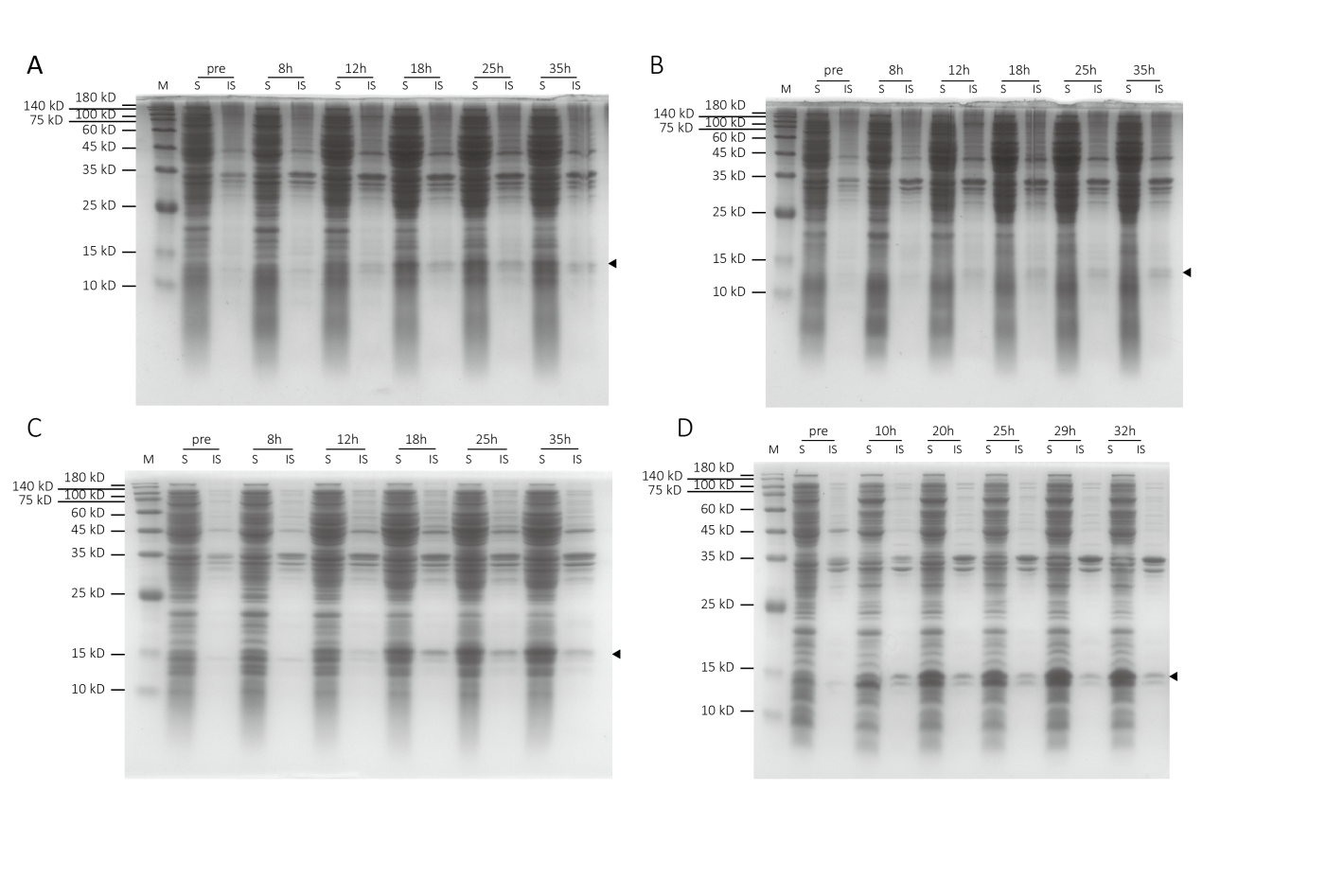


**Supplementary Figure 6.** The expression of anti-GFP VHHs with different SPs in *E.coli* Origami2 (DE3) via auto-induction. (A) OmpA, (B) PelB, (C) L-AsPs II, (D) VHHs alone. S: soluble fraction; IS: insoluble fraction. The arrow heads pointed the position of anti-GFP VHHs.


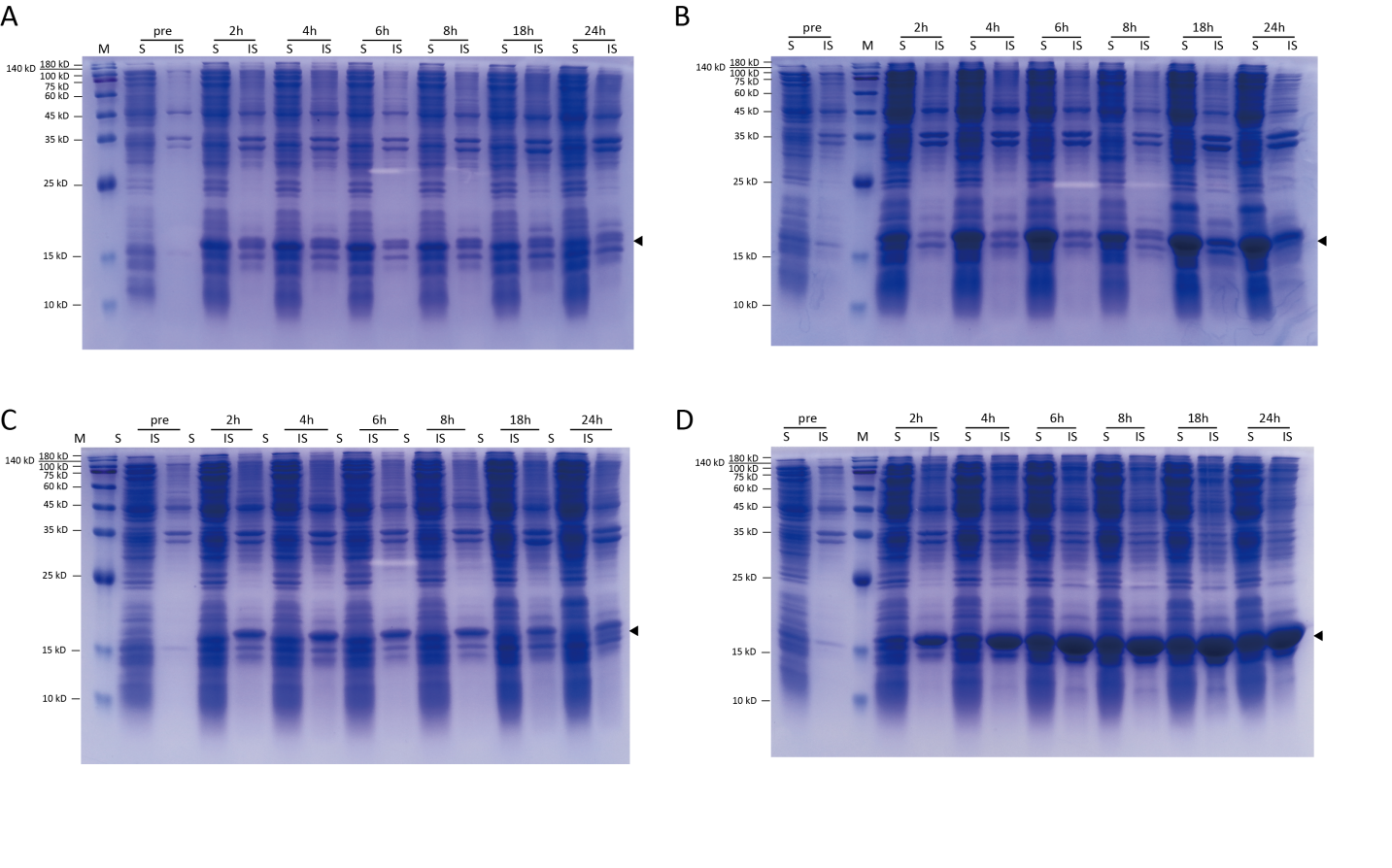


**Supplementary Figure 7.** The expression of anti-GFP VHHs with different SPs in *E.coli* BL21(DE3) via IPTG induction (repeat 2). (A) OmpA, (B) PelB, (C) L-AsPs II, (D) VHHs alone. S: soluble fraction; IS: insoluble fraction. The arrow heads pointed the position of anti-GFP VHHs.


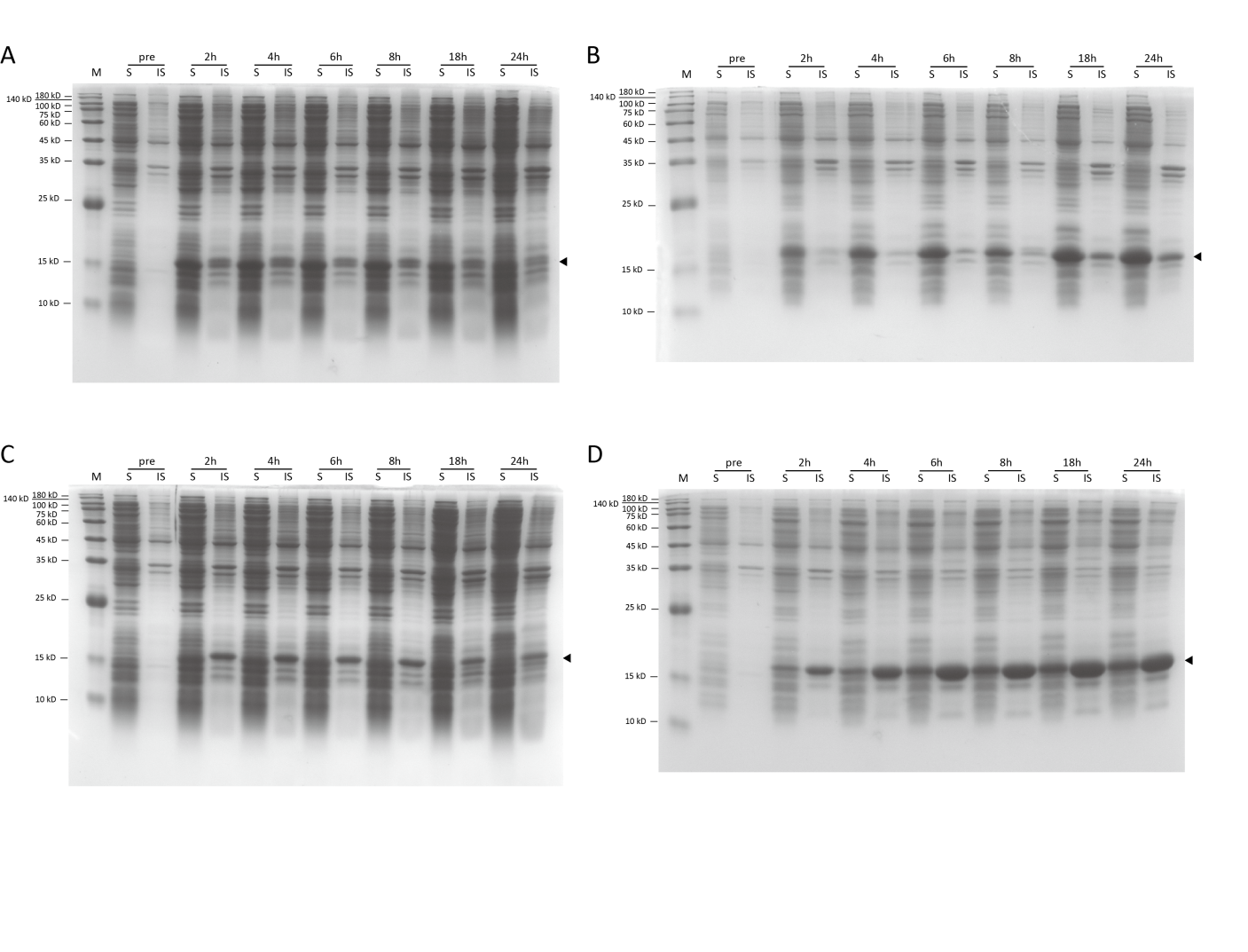


**Supplementary Figure 8.** The expression of anti-GFP VHHs with different SPs in *E.coli* BL21(DE3) via IPTG induction (repeat 3). (A) OmpA, (B) PelB, (C) L-AsPs II, (D) VHHs alone. S: soluble fraction; IS: insoluble fraction. The arrow heads pointed the position of anti-GFP VHHs.


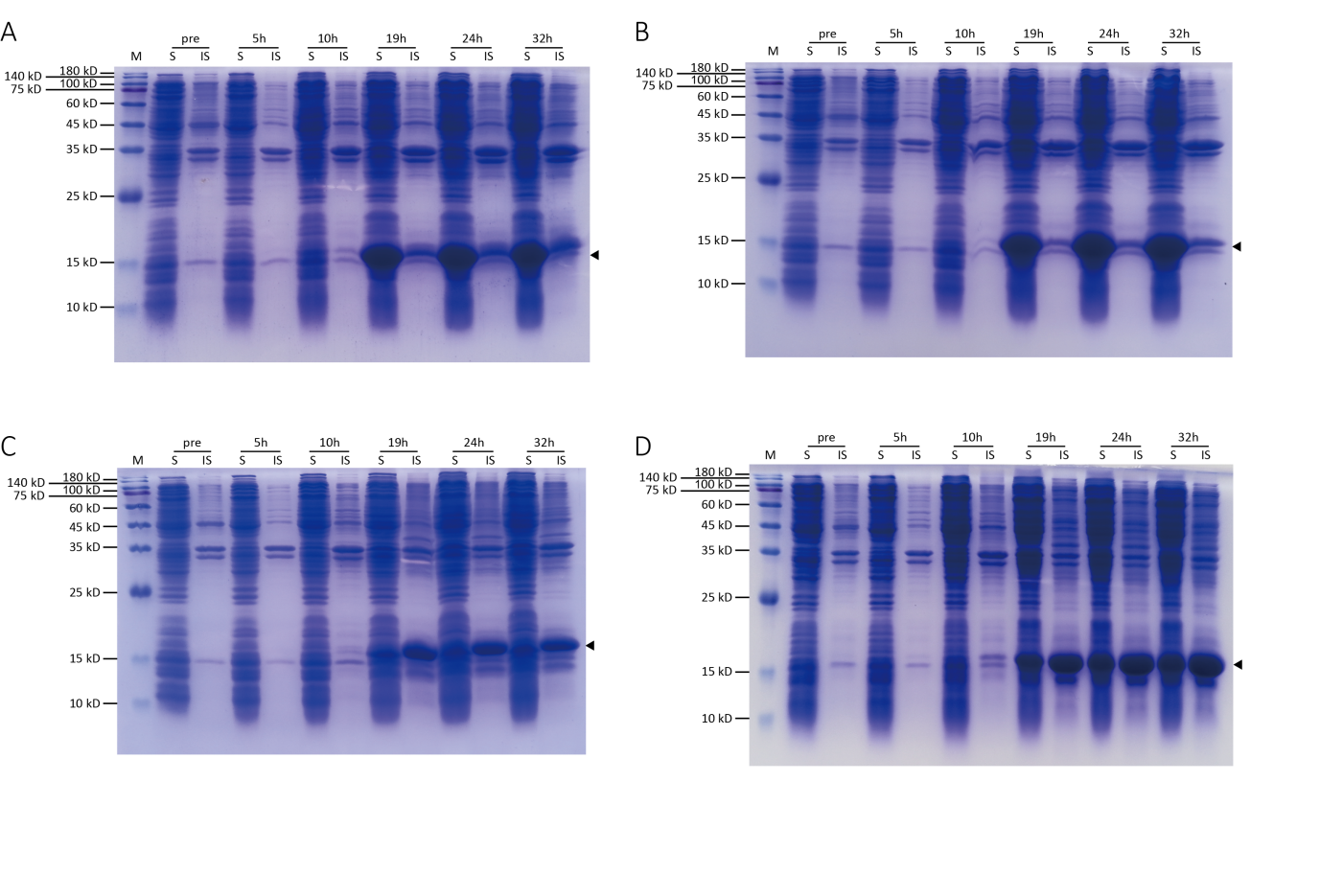


**Supplementary Figure 9.** The expression of anti-GFP VHHs with different SPs in *E.coli* BL21(DE3) via auto-induction (repeat 2). (A) OmpA, (B) PelB, (C) L-AsPs II, (D) VHHs alone. S: soluble fraction; IS: insoluble fraction. The arrow heads pointed the position of anti-GFP VHHs.


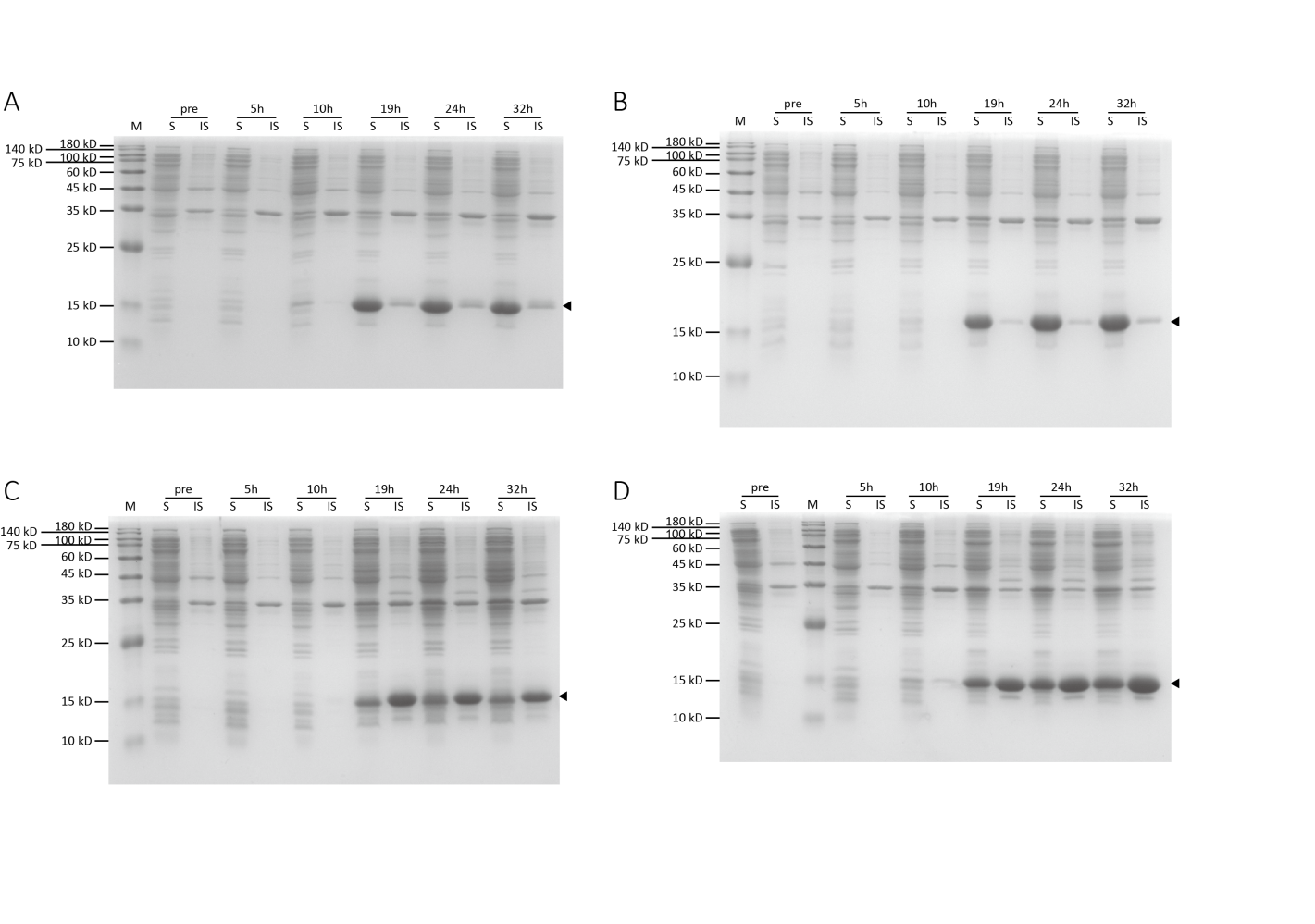


**Supplementary Figure 10.** The expression of anti-GFP VHHs with different SPs in *E.coli* BL21(DE3) via auto-induction (repeat 3). (A) OmpA, (B) PelB, (C) L-AsPs II, (D) VHHs alone. S: soluble fraction; IS: insoluble fraction. The arrow heads pointed the position of anti-GFP VHHs.
